# Designing miRNA-Based Synthetic Cell Classifier Circuits Using Answer Set Programming

**DOI:** 10.3389/fbioe.2018.00070

**Published:** 2018-06-22

**Authors:** Katinka Becker, Hannes Klarner, Melania Nowicka, Heike Siebert

**Affiliations:** ^1^Department of Mathematics and Computer Science, Freie Universität Berlin, Berlin, Germany; ^2^IMPRS-CBSC, Max Planck Institute for Molecular Genetics, Berlin, Germany

**Keywords:** Boolean modeling, Answer Set Programming, synthetic biology, miRNA profiles, cell classifier, breast cancer

## Abstract

Cell classifier circuits are synthetic biological circuits capable of distinguishing between different cell states depending on specific cellular markers and engendering a state-specific response. An example are classifiers for cancer cells that recognize whether a cell is healthy or diseased based on its miRNA fingerprint and trigger cell apoptosis in the latter case. Binarization of continuous miRNA expression levels allows to formalize a classifier as a Boolean function whose output codes for the cell condition. In this framework, the classifier design problem consists of finding a Boolean function capable of reproducing correct labelings of miRNA profiles. The specifications of such a function can then be used as a blueprint for constructing a corresponding circuit in the lab. To find an optimal classifier both in terms of performance and reliability, however, accuracy, design simplicity and constraints derived from availability of molcular building blocks for the classifiers all need to be taken into account. These complexities translate to computational difficulties, so currently available methods explore only part of the design space and consequently are only capable of calculating locally optimal designs. We present a computational approach for finding globally optimal classifier circuits based on binarized miRNA datasets using Answer Set Programming for efficient scanning of the entire search space. Additionally, the method is capable of computing all optimal solutions, allowing for comparison between optimal classifier designs and identification of key features. Several case studies illustrate the applicability of the approach and highlight the quality of results in comparison with a state of the art method. The method is fully implemented and a comprehensive performance analysis demonstrates its reliability and scalability.

## 1. Introduction

With the ongoing development of sophisticated engineering methods for biological components, the benefits of synthetic biology for medical applications are discussed more and more (Kis et al., 2015). Engineered gene circuits show promise in diagnostics and treatment (Kis et al., 2015; Slomovic et al., 2015). An emerging approach combining both tasks is targeting cells selectively with so-called classifier circuits. A cell classifier circuit is a synthetic biological circuit which may be delivered to a living cell on a plasmid or viral vector and then is capable of recognizing the cell state (e.g., healthy or diseased) based on its molecular fingerprint (e.g., using miRNA profiles). Depending on the classification result a controlled production of a desired output may be triggered and lead to cell apoptosis. In case of diseases such as cancer, where the heterogeneity and diversity of cells poses one of the major challenges, cell classifiers may provide a future avenue to more effective and non-toxic treatments based on personalized miRNA profiles (Heng et al., 2011; Leva and Croce, 2013; Mohammadi et al., 2017).

Rational design of synthetic biological systems is a complex task. Assembly of an *in silico* designed circuit in the laboratory is costly and time-consuming. Here, mathematical modeling is highly valuable for the design process because it allows the formalization of demands and the computation of optimal solutions within the design space (Teo et al., 2015; Mohammadi et al., 2017). Since many of the biological building blocks for synthetic circuits, and in particular classifiers, are geared toward steep response profiles to ensure robust performance and assembled as logic gates (Singh, 2014; Siuti et al., 2015), Boolean modeling approaches are well-suited for this task. However, the logical formalization often remains implicit and the computational capabilities within the Boolean framework remain largely unused (Xie et al., 2011; Moon et al., 2012; Mohammadi et al., 2017).

In this article, we show the potential of formal methods, in particular Answer Set Programming (ASP), in the context of classifier design. Although the underlying ideas are broadly applicable, we tailor our implementation to the task of processing miRNA profiles to distinguish between healthy and cancerous cells. In this context, a classifier is represented by a Boolean function that, given as input a discretized miRNA profile, outputs the binary cell state encoding healthy or diseased. A similar problem is considered in work by Mohammadi and colleagues (Xie et al., 2011; Mohammadi et al., 2017). They developed a transcriptional/post-transcriptional synthetic regulatory circuit capable of performing a desired task. In addition, they formulate constraints for the classifier design that need to be satisfied so that the classifiers become biologically viable. Constraint satisfaction complicates the search for optimal designs significantly, so that approaches usually employ heuristics for exploring the design space (Mohammadi et al., 2017). However, heuristic methods do not guarantee that globally optimal solutions are found and so there is no guarantee that much better solutions are not overlooked.

Exploiting the potential of ASP as a powerful solver for constraint satisfaction problems, we present an approach that allows to compute globally optimal classifiers that satisfy all given constraints. In the hierarchy of optimization criteria, the strongest emphasis is placed on classifier accuracy in terms of classification errors followed by circuit simplicity in terms of number of inputs and utilized gates. The computational power of the approach allows us to calculate all optimal solutions that can be further distinguished using scores relating the discrete results to the continuous data. Not least, comparison of those optimal solutions can uncover key classifier features as well as highlight variability in design. After describing the formalization and strategies for solving the problems, we present our results for five breast cancer case studies and compare them with the output of the heuristic approach of Mohammadi et al. (2017). To assess general applicability, we present a performance analysis of our method based on synthetic data. In the discussion we address issues beyond the Boolean abstraction that can be tackled in order to evaluate and increase the quality of solutions.

## 2. Methods

**Figure 1:**
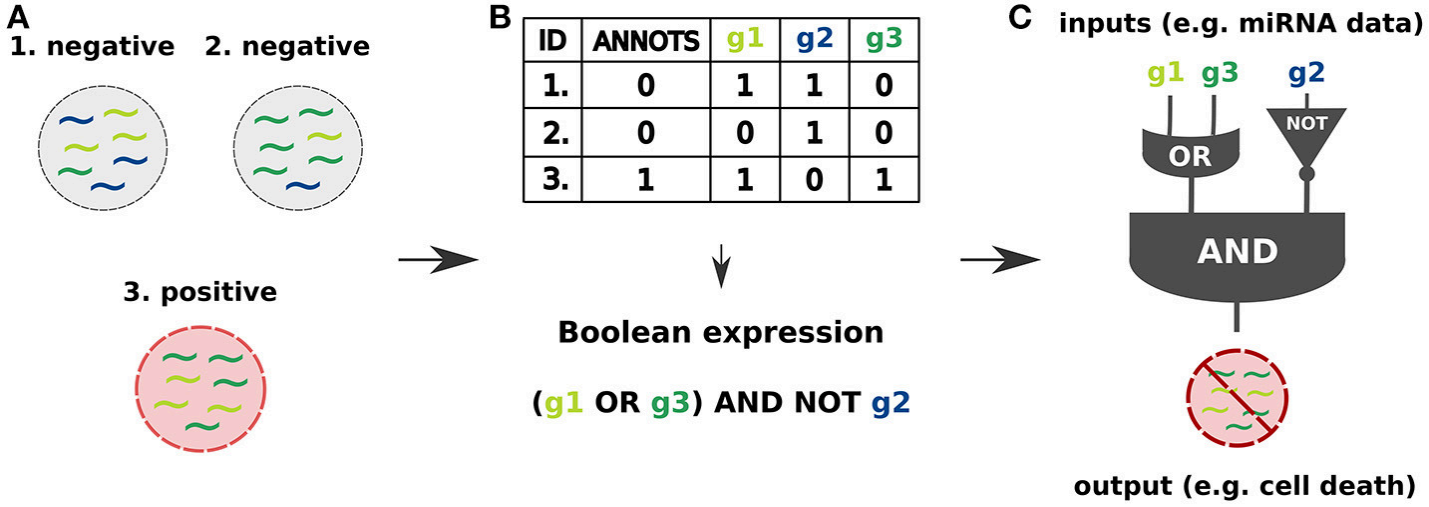
Cell classifier circuits are synthetic logic circuits capable of sensing endogenous molecular signals (inputs) in a living cell, classifying them as type-specific signals and triggering a desired response (output) based on the classification result (Xie et al., 2011; Mohammadi et al., 2017). **(A)** The inputs may be specified as miRNA expression profiles identifying the cell state as healthy or diseased. **(B)** Binarization of continuous miRNA levels, characterizing them as high or low, allows to combine them into logic gates and define a Boolean function which allows to classify the cell state. **(C)** The input signals are processed by a synthetic regulatory network capable of triggering the response, which may be a controlled production of a desired RNA or protein output leading to a cell apoptosis.

Similar to electronic circuits, synthetic gene circuits are designed in terms of logic gates (Singh, 2014; Siuti et al., 2015) within a Boolean framework. Typically they consist of disjunctions (OR gate), conjunctions (AND gate) and negations (NOT gate) of input signals that are interpreted as Boolean variables. A classifier is then a Boolean function that yields for the different input combinations an output signifying the classification (e.g., healthy or diseased). An example of a simplified classifier design process is given in Figure 1.

Here, we focus on miRNA-based cell classifiers for cancer datasets where input signals are miRNA expression levels that are binarized into two qualitative levels: *High* (1) and *Low* (0) expression, that is, above and below a given threshold value. The aim of the classifier is to determine whether a given sample is healthy or cancerous by processing the corresponding miRNA fingerprint of the sample. This setup is similar to a supervised machine learning problem because each sample is labeled by the cell state. Mathematically, the challenge is to construct a Boolean function that distinguishes the two types of samples. The function should return *True* for inputs that correspond to cancerous samples and *False* otherwise. The dataset is not required to and usually does not cover all possible inputs, that is, all miRNA that may occur in the cell, and all combinatorically possible input combinations. Whatever the classifier returns for unseen combinations of miRNAs is irrelevant, although it may naturally be used to predict the states of new samples.

If the data is consistent, that is, the same profile has not been observed to characterize both a healthy and a diseased cell, and does not cover all input combinations then there will be more than one classifier. In practice, it is then interesting to choose from the set of mathematically feasible classifiers those that are biologically feasible while minimizing a cost function that represents the actual cost of assembling the classifier in a laboratory.

### 2.1. The programming environment

To implement our approach to classifier design we made use of Answer Set Programming (Lifschitz, 2008), a form of declarative programming. Declarative programming, in contrast to imperative programming, allows to define only a computational logic of the program without explicitly specifying a strategy to solve the problem. In case of ASP a detailed problem description including the data, the constraints on a classifier and the optimization strategy is encoded as logical rules. Those logical rules, called the ASP program, are then instantiated by an ASP grounder and solved by an ASP solver. Various systems for grounding and solving are available. For our calculations we use the grounder *gringo* and the solver *clasp*, both part of the Potsdam Answer Set Solving Collection *Potassco* (see Data and Software Availability). For more information about the ASP encoding see the Supplementary Material.

**Figure 2:**
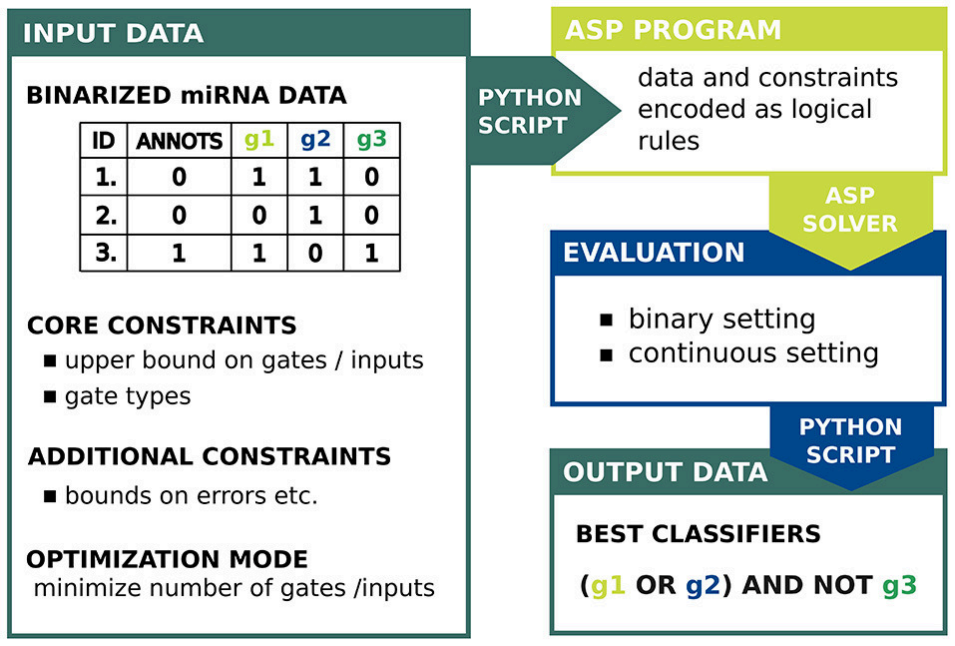
The scheme for our ASP-based approach to synthetic gene circuit design.

The full approach is implemented in a set of Python scripts, available on GitHub with a detailed description and a manual. The script *classifier.py* allows to convert the input data into the ASP program, call the *Potassco* grounder and solver and create images of the classifiers as directed graphs. Our approach comprises also an evaluation of biochemical capabilities of a designed circuit to predict the efficacy of response triggering (*scores.py*). To estimate the real-world performance we implemented classification scores proposed by Mohammadi et al. (2017). In the following we describe the setting and all steps in the proposed workflow. A schematic view of our approach is given in Figure 2.

### 2.2. The data: miRNA expression profiles

**Figure 3:**
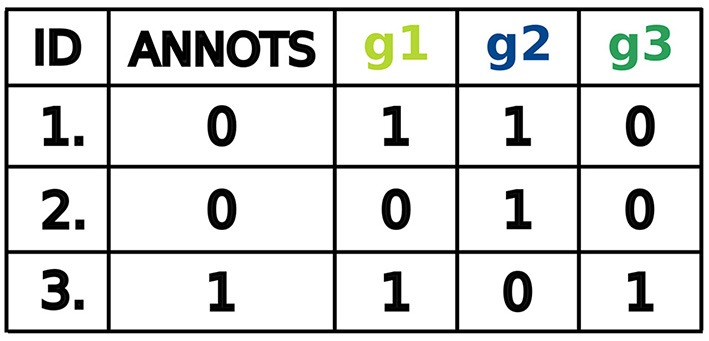
Example data set for 3 samples: 2 negative and 1 positive.

For our purposes, a dataset is a table of binarized miRNA expression profiles for different samples. The first column referenced as ID contains numbers uniquely identifying the samples. The second column referenced as Annots states for each sample its annotation and indicates whether the sample is cancerous or not. Here, samples are labeled by a binary variable, that is, positive samples are annotated as 1 and negative samples as 0. The next columns represent the binarized miRNA levels in each sample. Every column is labeled by a letter g followed by an integer number that uniquely identifies a miRNA. Therefore, each row includes a miRNA expression profile, that is, a set of binarized miRNA levels, corresponding to the samples. High levels of miRNAs are labeled as 1 and low levels as 0. A running example is given in Figure 3.

Naturally, data discretization has to be handled with care since results will depend on the chosen thresholds. A variety of different discretization methods are available, see an example work by Gallo et al. (2016), and the choice should depend on characteristics of the dataset and the ultimate purpose of the discretization. We will comment on this issue in more detail in the discussion.

### 2.3. Boolean classifiers

In our context, a *classifier* is a Boolean function *f*:{0, 1}^*n*^ → {0, 1}, where *n* is at most the number of considered miRNAs in a profile. We consider *f* to be in Conjunctive Normal Form (CNF), that is, it is a conjunction of clauses where each clause is the disjunction of negated or non-negated inputs. A perfect classifier is a Boolean function that allows to accurately separate samples into two groups (e.g., healthy and diseased) based on Boolean inputs (e.g., miRNA levels). The Boolean output value for each sample must be equal to the sample annotation value in the Annots column. Classifiers which cannot separate samples perfectly, that is, some samples annotated as negative are classified as positive (false positive errors) or some samples annotated as positive are classified as negative (false negative errors), are called imperfect classifiers.

An example of a perfect classifier for the dataset shown in Figure 3 is:

(g1∨g3)∧¬g2

where ∧, ∨ and ¬ represent logical conjunction, disjunction and negation, respectively. This classifier consists of two clauses, namely (*g*1 ∨ *g*3) and ¬*g*2, which we call *gates* in the context of synthetic biology. The difference between two gates is that the second one has only one input and the first two. Also, the second gate has a negated input while the first consists only of positive inputs. In terms of classifier design the function may be interpreted as follows: miRNA-g1 or miRNA-g3 should be highly expressed and miRNA-g2 should be low expressed in the sample to classify the sample as positive. Then a sample is classified as positive in three cases: (1) both miRNA-g1 and miRNA-g3 are highly expressed and miRNA-g2 is low expressed, (2) miRNA-g1 is highly expressed, miRNA-g3 and miRNA-g2 are low expressed and (3) miRNA-g3 is highly expressed, miRNA-g1 and miRNA-g2 are low expressed.

### 2.4. Classifier constraints

**Figure 4:**
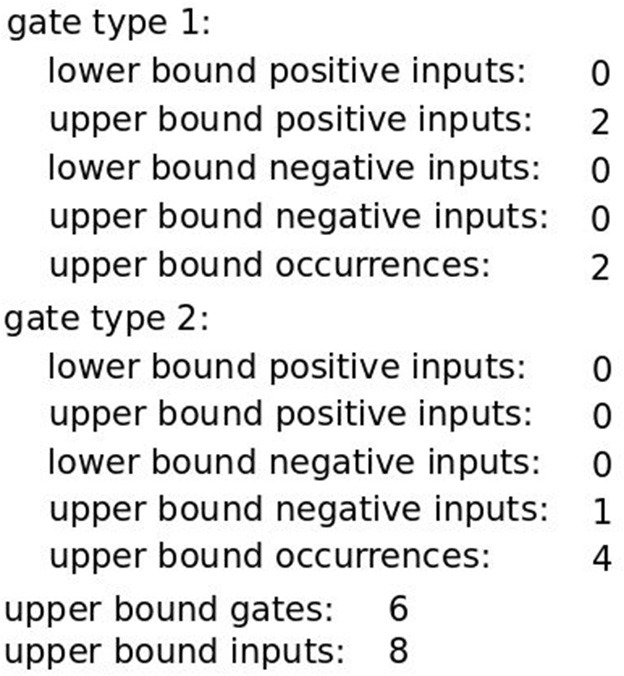
A full set of core classifier constraints. Here, a classifier may consist of up to 6 gates with overall up to 8 inputs. Gates are either of Type 1 (OR gate) or Type 2 (NOT gate) where the first may have only non-negated inputs while the second may only be a single negated input.

Differences in the structure of the Boolean function, for example, how the gates are formed, are relevant because they may result in classifiers that cannot be assembled in the laboratory or in classifiers that are very expensive to assemble. Based on the study of Mohammadi et al. (2017) we added constraints that capture the feasibility of constructing a synthetic circuit based on the Boolean function. As smaller and simpler classifiers are easier to assemble an obvious first step is to constrain the overall number of inputs and gates that appear in the classifier by introducing upper bounds on both of them. Additionally, only two types of logic gates may be assembled in the laboratory (Mohammadi et al., 2017). Thus, the allowed *gate types* have to be defined. The gate type description consists of 5 different bounds: lower and upper bounds for positive and negative inputs and an upper bound on the total number of occurrences of the gate in the classifier. The set of above-mentioned constraints we call the *core constraints*. An example of a full set of classifier constraints allowing solutions viable for construction in the lab is given in Figure 4.

The core constraints may in many cases be extended to specific needs without much effort. Here, we describe two that are implemented in our software.

First, it may easily happen that the chosen constraints are not satisfiable for a given dataset, that is, the perfect classifier does not exist. This may be caused by the diversity between cancer samples, but also by experimental artifacts or data preprocessing errors, for example, in the data discretization step. In such case, we can search for an imperfect classifier allowing misclassification of a certain number of samples. Thus, we introduced two additional constraints: upper bounds on false positive and false negative errors. The optimization procedure for imperfect classifier optimization we call *constraint relaxation*. A detailed description is given in the next section.

It is worth considering which type of error we may accept or even neglect. If the desired response is to cause the cell apoptosis, a false negative error results in a wrong diagnosis and a cancerous cell survives. In case of a false positive error a healthy cell will be diagnosed as diseased and killed. In case of false negative errors we may avoid killing healthy cells increasing the misclassification expectancy for the diseased cells at the same time. The first presented case study, Breast Cancer *All*, illustrates the flexibility of our method for finding a feasible classifier in case of forbidding the false positives.

Second, we formulated the *unique-input constraint* that guarantees that miRNA inputs are not shared across different gates. Constraints of this type, that is, forbidding the use of certain combinations of miRNAs, might be relevant for increasing the robustness of the design against noise in miRNA levels.

### 2.5. Finding optimal classifiers

Once a feasible classifier is specified in terms of gate types and bounds on inputs and gates, it is usually of interest to find one that is optimal with respect to a given cost function. Putting the focus on finding structurally simple classifiers to facilitate construction, we propose the following optimization problems for finding a perfect classifier:

(Opt1) Minimize the number of inputs.
(Opt2) Minimize the number of gates.
(Opt3) Minimize the number of inputs followed by the number of gates.
(Opt4) Minimize the number of gates followed by the number of inputs.

The two problems (Opt3) and (Opt4) are bi-level optimization problems where the upper-level problem is solved first followed by the lower-level problem. Each of the four strategies may lead to a different classifier even for the same dataset and classifier constraints. Here, it might also be interesting to run both (Opt3) and (Opt4). Results can be evaluated and the final design chosen accordingly.

In the context of the ASP programming environment (see the Supplementary Materials) the strategies are implemented via minimizing weighted sums over all inputs and gates. Other optimization strategies may be formulated using specific weighted sums. In particular, it is possible to penalize the use of certain miRNAs as inputs, for example, if these miRNAs are known to interfere with other components. The weights can also be chosen to favor miRNAs whose average distance from the binarization threshold is large. Apart from decreasing sensitivity to the threshold choice, this might be beneficial in terms of robustness to expression level noise. We will discuss these possibilities later in more detail.

Note that we optimize globally, that is, all solutions (if found) are the best solutions among all other feasible solutions for a given dataset. In other words, if a given classifier is a solution of optimizing with strategy (Opt1) and it consists of 7 inputs then a classifier with less than 7 inputs for a given dataset under given constraints does not exist. In addition, the ASP-based method allows to list all optimal solutions, if more than one exists. Analysis of commonalities and differences of the optimal classifiers can then provide additional insight into the importance of specific inputs or circuit modules for the classification.

However, for some datasets it is impossible to find an optimal solution satisfying all constraints. As mentioned before, to tackle that issue we incorporated an optimization procedure for finding imperfect classifiers (but respecting the *core constraints* presented in Figure 4) by allowing a certain number of false positive and false negative errors using *constraints relaxation*. In practice, we first apply an optimization procedure for finding a perfect classifier without allowing any errors. If a solution does not exist, we relax the constraints stepwise by increasing the upper bound values for errors ultimately allowing as many errors as necessary to find an imperfect classifier. Depending on the application, we can decide to only allow errors of a specific type or increase the upper bounds of both types. For the presented case studies we first looked for a classifier allowing up to 1 false negative and 0 false positives, then up to 0 false negatives and 1 false positive, 1 false negative and 1 false positive etc. In case of finding different solutions allowing the same number of errors in total we choose a solution with less inputs. However, the procedure may be easily changed. We present the flexibility of the method in two case studies. In both, we first search for a classifier using the procedure described above. In the Breast Cancer *All* case study we additionally forbid one type of error and search for a classifier increasing the number of the second type. Depending on the application we may need to forbid one type of error. We discuss it in detail when presenting the case study. In the *ER*+ *Her*− case study we look for a first solution with the standard procedure and then look for another classifier by further increase of the bounds on errors. In case when the first found classifier is not short enough or we cannot include one of the miRNAs it may be useful to extend the set of feasible classifiers. Regardless of the specific treatment of errors, classifier accuracy is put on the highest level of the optimization, followed by classifier simplicity as described above.

Usually, optimal solutions are not unique. There may be several optimal designs that differ in the miRNAs that are used or in the way inputs are assigned to gates. In those cases it might be insightful to enumerate all optimal solutions, for example to investigate common structural features, or simply to ask which miRNAs do or do not appear in optimal classifiers. However, when interested in this feature we need to take care of symmetries generated in the process of finding the classifiers. ASP allows for isomorphic classifiers to be counted as different solutions. Gates are for example assigned an integer identifier which we need in order to determine which inputs belong to which gate. Any permutation of assigning IDs to gates will therefore be counted as a separate solution. Breaking symmetries is an involved topic of its own. Its importance lies in the fact that the number of symmetric solutions may explode and seriously hamper the calculation of all solutions. However, in our applications we can still easily solve this problem through a post-processing step. Within a set of optimal solutions returned by the ASP solver we first sort the gates of each classifier by the IDs of inputs. Then we rewrite all the solutions by assigning to each gate an ID in ascending order preserving the original gate to input relation. The input and gate IDs are then ordered identically for all the isomorphic solutions in each class what makes them indistinguishable. An arbitrary solution from such a subset can be picked as a representative. This procedure is illustrated in the first case study presented in section 3.2

### 2.6. Classifier evaluation

To assess the classifiers resulting from the optimization procedure we incorporated in our workflow an evaluation step. We distinguish two settings for the classifier assessment: Boolean and continuous. In the Boolean setting we consider discretized datasets and classifiers, that is, Boolean functions, to evaluate how well the function separates samples for a given dataset. In a continuous setting we estimate how well a classifier will perform in a setting closer to reality. Here, we adopt an approach by Mohammadi et al. (2017), which allows us to also compare our results directly. It is based on the performance of a classifier represented by a mechanistic model of the circuit represented by Hill equations derived from the Boolean classifier on real-valued miRNA data (Mohammadi et al., 2017).

To asses classifiers in the Boolean setting we calculate the false positive and false negative rates. Both rates allow to estimate the expectancy that a sample may be misclassified and how well a classifier performs in a context of classifying the data.

To evaluate classifiers in the continuous setting we calculate, as mentioned, the classifier scores proposed by Mohammadi et al. (2017). The authors developed a mathematical model of a synthetic regulatory network corresponding to the circuit assembled in the laboratory. The model is represented by a set of Hill equations and describes, for a given Boolean classifier, an input-output relation, where the inputs are the real-valued miRNA expression levels and the output is a concentration of a protein or another desired compound produced by the circuit. Based on the available information on biochemical reaction rates the model allows to predict the circuit output concentration for the continuous miRNA data and a Boolean classifier with respect to the choice of output binarization thresholds. In other words, we use the model to estimate whether the concentration of the output will be sufficient to cause, for example, cell death. For more details of the procedure see the article of Mohammadi et al. (2017).

The first score (*S*_*AUC*_) that is considered with the help of the continuous model, the area under the ROC curve (AUC), predicts how well the classifier responds based on different thresholds for the circuit output concentration. Additionally, Mohammadi et al. (2017) distinguish two different margins: the average margin (*Ma*) states the overall ratio between the output in the positive vs. the negative sample class and shows how well all samples in the dataset are separated by the output concentration. The worst margin (*Mw*) is the smallest output ratio among any pair of positive and negative samples and helps to capture the outliers. The second score (*S*_*m*_) is represented by a weighted sum of these margins:

$$S_{m}=\lambda Ma+\left(1-\lambda \right)Mw$$

where λ ∈ [0, 1] is a weight that specifies the contribution of the particular margins. For the breast cancer data sets we used λ = 0.5 (assuming that both margins are equally relevant) applied also by Mohammadi et al. (2017) in all studies. Here, we extend our procedure for finding optimal classifiers described in the previous section with these two scores. To choose between classifiers that are all optimal, firstly, with respect to the total number of errors and, secondly, with respect to the number of inputs followed by the number of gates, we first look for classifiers with the highest *S*_*AUC*_ and then with the highest *S*_*m*_. We preserve all details of the strategy proposed by Mohammadi et al. (2017) to make the presented results comparable.

As an additional evaluation of our approach, we perform, if the data permits, cross-validation to test the predictive power of the calculated optimal classifiers when facing entirely new samples. We illustrate that for the largest case study dataset below, where we performed a 3-fold cross validation.

### 2.7. The benchmarking: data generation

To have a broad picture of the performance of our method we tested our approach on simulated datasets. Here, we describe how the datasets were prepared.

We generated random 0-1 matrices, with each entry independent and 0-1 equally likely to occur, of all dimensions starting from 10 by 10 going up to 500 by 500 where the step-sizes of increasing rows and columns are both 10. The benchmark consists therefore of 50 × 50 = 2, 500 binary matrices representing miRNA expression data sets. For each matrix we generated *annotation classifiers* that we used to decide which of the samples are going to be labeled as positive and which as negative. For each of the four optimization strategies as well as for the problem of finding a non-optimized classifier, we measured the time to compute the first solution classifier that satisfies the *core constraints*.

Here, we propose two setups for the data generation. In *Setup 1*, we randomly created *annotation classifiers* that satisfy the *core constraints* by choosing, with equal probabilities, the existing gates and inputs. This setup guarantees the existence of a solution to each ASP problem.

In *Setup 2* we constructed the *annotation classifiers* using a binomial distribution. We defined the maximal number of gates as ⌊*n*/10⌋ and the maximal number of inputs per gate as 5. For a given data matrix we then “tossed a fair coin” for the existence of each gate and each of its possible inputs. The assignment of miRNAs to inputs was done with equal probabilities. Of course, the two parameters (10 and 5) are somewhat arbitrary. The intention was to find out how the algorithm performs when it is not guaranteed that there is a solution, which contrasts Setup 1.

### 2.8. The cross-validation

In addition to the run-time analysis for our ASP-based implementation we also assessed the quality of the predictions of the classifiers. We decided to record the generalization error of a 10-fold cross-validation for Setup 1 (guaranteed existence of solution), for finding feasible and optimal solutions. The cross-validation was conservative in that we treated time-outs as false predictions.

For the cross-validation the rows of each matrix of each data point were divided into 10 parts of equal size. For each tenth we built a classifier based on the 9 remaining parts only. All mismatches between the resulting classifier and the given *annotation classifier* on the test set were counted and added for each tenth. This sum, divided by the sample size, is the generalization error: the fraction of samples that were incorrectly predicted in all 10 runs. Note that, as a consequence, the validation procedure performs 10 times the calculations of a benchmark.

All computations, for both the benchmarking and the cross-validation, were performed on a Linux AMD64 with 2.83 GHz and 32 GB of memory.

## 3. Results

To test and evaluate our approach in application we present a case study with five datasets also considered by Mohammadi and colleagues, allowing a subsequent comparison with their results. We complement the case study with a performance analysis using synthetic data.

### 3.1. Case studies: breast cancer data

The following breast cancer datasets have been presented by Farazi et al. (2011) and preprocessed by Mohammadi et al. (2017) via miRNA expression normalization, aggregation of similar miRNAs and discretization into two levels: high expression and low expression. The breast cancer dataset referenced as *All* includes all samples and consists of four subsets for different subtypes of breast cancer. We present a summary description of the data with the number of miRNAs taken into account and binarization thresholds applied for the discretization of the continuous data in Table 1.

**Table 1:** Breast cancer dataset description: overall number of samples, number of positive and negative samples, number of miRNAs taken into account and binarization threshold applied for data binarization.

**Subtype**	**Samples**	**Positive**	**Negative**	**miRNA**	**BinThreshold**
All	178	167	11	478	250
Triple-	82	71	11	456	250
Her2+	86	75	11	438	1,250
ER+ Her−	32	21	11	392	1,250
Cell Line	17	6	11	375	50

We searched for classifiers for the breast cancer *All* dataset to separate cancerous from healthy samples and for each breast cancer subset to separate cancerous samples of each subtype from healthy samples. For each dataset we first applied the (Opt3) optimization mode (minimize the number of inputs followed by the number of gates) and searched for a perfect classifier respecting the *core constraints* presented in Figure 4. For one dataset called *Cell Line* a perfect single-input classifier was found, that is, the smallest dataset may be perfectly separated by only one miRNA. However, for four other datasets (*All, Triple-, Her2*+, *ER*+ *Her*−) perfect classifiers do not exist. Thus, we applied the constraints relaxation procedure.

### 3.2. Breast Cancer All

**Figure 5:**
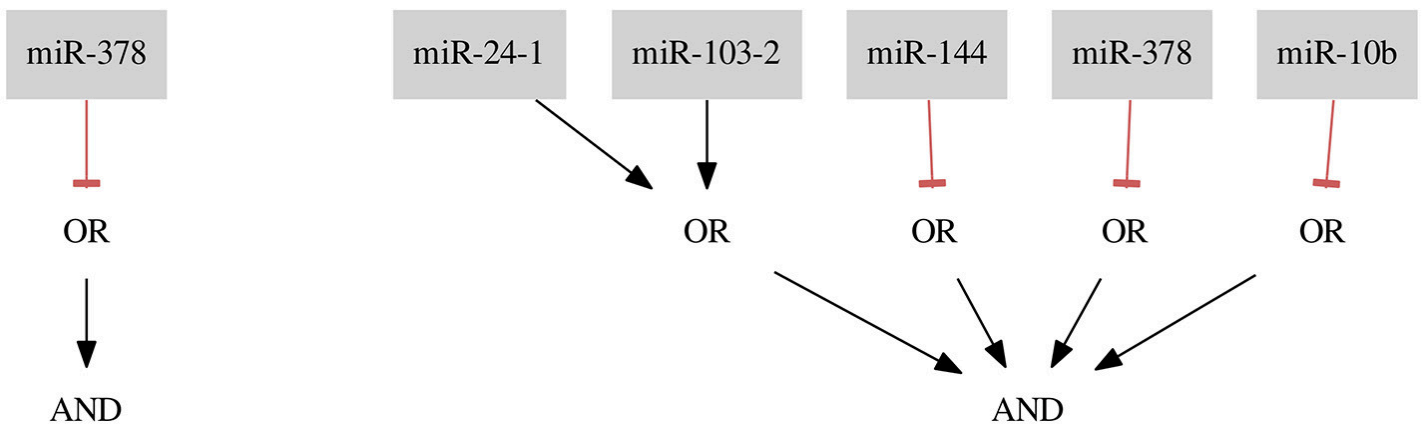
Classifiers for Breast Cancer All data set.

For the combined Breast Cancer *All* dataset we found two classifiers presented in Figure 5.

The first classifier consists of only one gate and one input *miR-378* and results in misclassification with four false negative (FN rate = 0.02) and three false positive (FP rate = 0.27) errors. The classifier is the shortest possible classifier and is easy to assemble in the laboratory. However, it is worth considering whether one input classifiers are reliable enough to tackle the cancer cell diversity.

As mentioned before, it is also worth considering whether one type of error is less desirable or entirely forbidden. Here, we present results of optimization where we do not allow the false positives to occur. In this case, we find 6 optimal solutions. However, all of them are just artifacts of the implementation, namely being isomorphic to the classifier presented in Figure 5. If we compare the here presented classifier (miR-24-1 ∨ miR-103-2) ∧ (¬ miR-144) ∧ (¬ miR-378) ∧ (¬ miR-10b) with one of 5 remaining solutions, for example, (miR-24-1 ∨ miR-103-2) ∧ (¬ miR-378) ∧ (¬ miR-10b) ∧ (¬ miR-144) it is clearly visible that these solutions differ in the order of miRNA IDs assigned to the three NOT gates. All 6 isomorphic solutions differ, in fact, in the permutations of the 3 different IDs assigned to 3 NOT gates and belong to the same isomorphism class. We process the solution set to eliminate these copies as described in section 2.5. Ultimately, we find only one isomorphism class for this dataset. Although in this case one is able to easily distinguish the isomorphic solutions without employing an automated post-processing step it may happen that the computation results in a few isomorphism classes (e.g., in the case study Breast Cancer *Triple-* we find more than one class) and the classifiers consist of many inputs we may receive even thousands of isomorphic solutions. Then, using an automated approach to the solution scanning is inevitable.

Both classifiers presented in Figure 5 share the same gate and the same input *miR-378*, which is supposed to be down-regulated in the sample. The study of Farazi et al. (2011) describes *miR-378* as low expressed and confirms that the use of *miR-378* as a potentially down-regulated marker in a classifier is reasonable. Also an unrelated study shows that, e.g., *miR-144*, which occurs in the second classifier, is expected to be down-regulated in breast cancer (Pan et al., 2016).

**Figure 6:**
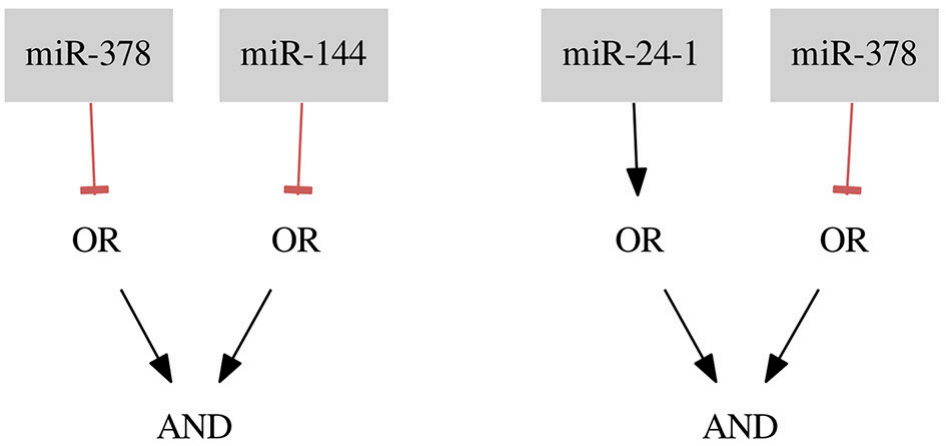
Classifiers for the breast cancer *Triple-* data set.

For the largest dataset we also performed a 3-fold cross-validation. The folds consist on average of 56 positive samples and only 4 negative samples. We divided the dataset in 3 almost equal subsets (the subset size differs in at most 1 sample) without taking an even distribution of positive and negative samples between subsets into account. For all folds it was necessary to apply the constraints relaxation procedure. The classifiers result on average in classification with FN rate = 0.01 and FP rate = 0.56 (FN occurrence average = 1, FP occurrence average = 3). The results show that our method was able to classify the positive samples almost perfectly. The very high FP rate may be a result of a very imbalanced division of negative vs. positive samples. We address the influence of imbalanced datasets on the results in the discussion. The cross-validation resulted in 2 different classifiers: (¬ miR-144) and (¬ miR-10b) AND (¬ miR-193a-5p). Both *miR-144* and *miR-10b* appeared in the second presented classifier (Figure 6) and both were marked as down-regulated in different studies (Farazi et al., 2011; Pan et al., 2016). However, also *miR-193a-5p* is marked as down-regulated by Farazi et al (Farazi et al., 2011). Thus, the choice of miRNAs seems to be reasonable.

### 3.3. Breast cancer Triple-

For the breast cancer *Triple-* dataset two classifiers were found. The first classifier consists of two gates of Type 2 (NOT gates) and the second classifier consists of one gate of Type 1 and one of Type 2. Both classifiers, shown in Figure 6, are the results of allowing 3 false negatives and 2 false positives (FN rate = 0.04, FP rate = 0.18). To decide between those classifiers it should be discussed in detail whether there are samples which are more reliable for the classification process or which type of gate is more desired. This choice makes our method flexible regarding particular experiments and datasets.

*miR-378* is marked as down-regulated in a study by Farazi et al. (2011). *miR-24-1* is described as up-regulated (Roscigno et al., 2017) and, as mentioned before, *miR-144* is described as down-regulated in breast cancer (Pan et al., 2016). Again, the proposed classifiers seem to be reasonable.

### 3.4. Breast cancer Her2+

**Figure 7:**
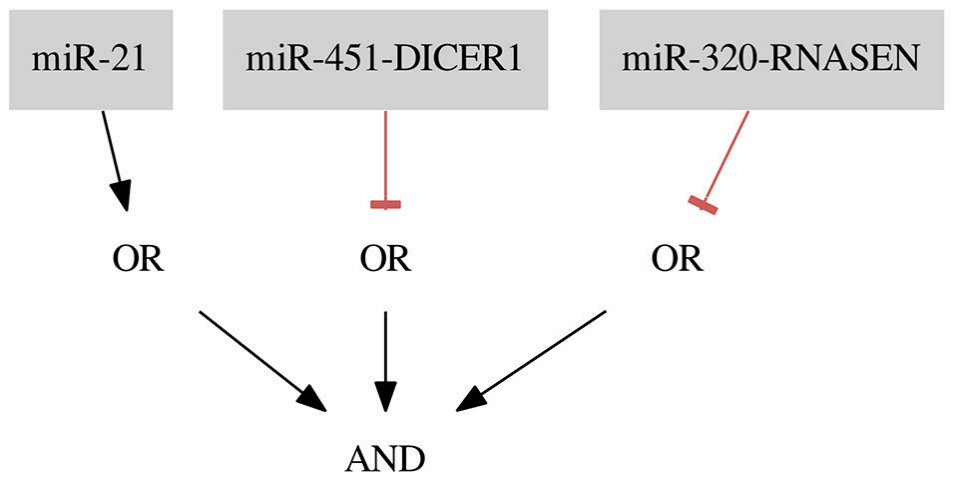
Classifier for the breast cancer *Her2*+ data separating samples with one false positive error.

The resulting classifier for the dataset *Her2*+ (shown in Figure 7) consists of three inputs and three gates: one gate of Type 1 with one input and two gates of Type 2 (FN rate = 0.00, FP rate = 0.09).

*miR-451-DICER1* and *miR-320-RNASEN* are marked as down-regulated and *miR-21* as up-regulated in a study by Farazi et al. (2011).

### 3.5. Breast cancer ER+ Her−

**Figure 8:**
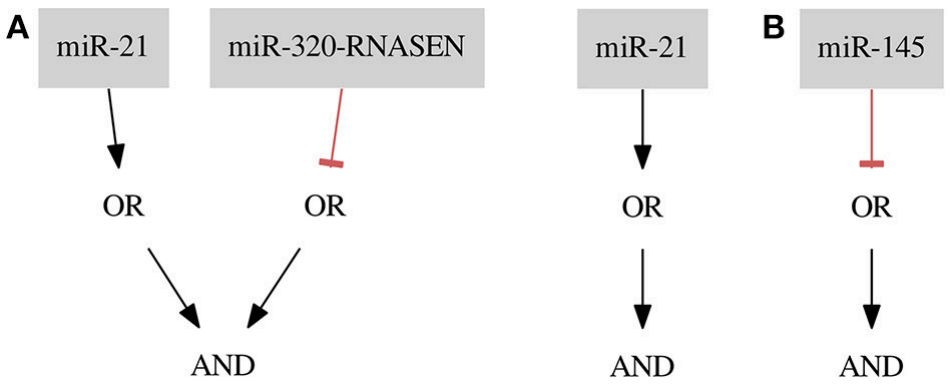
Results for the breast cancer **(A)** *ER*+ *Her*− and **(B)** *Cell Line* data sets.

For the *ER*+ *Her*− dataset we found one optimal classifier shown in Figure 6A on the left side. The classifier consists of two gates of both types and two inputs resulting in a classification with two false positive errors (FNrate = 0.00, FPrate = 0.18). Here, we present an additional classifier with only one input forming a gate of Type 1 resulting in a classification with three false positive errors (FN rate = 0.00, FP rate = 0.27) shown in Figure 8A on the right side. In this case we were able to obtain a shorter classifier relaxing the bounds on the number of false positives by only one additional error. It is worth considering whether the one misclassified sample is reliable or if we could neglect it and build a simpler classifier. Also, in case one of the miRNAs cannot be included in the classifier or the classifier consist of too many inputs it may be worth to further increase the bounds on errors. Note that the first classifier consists of only one gate and the same gate is a part of the second classifier.

*miR-21* is marked as up-regulated and *miR-320-RNASEN* as down-regulated by Farazi et al. (2011).

### 3.6. Breast cancer cell line

For the Cell Line dataset the optimization results in six perfect classifiers that consists of only one gate and one input each, where five of them are gates of Type 2 and only one is a gate of Type 1. That is, these classifiers distinguish cancerous from healthy samples based merely on the expression level of one miRNA (FN rate = 0.00, FP rate = 0.00). As an example we present a classifier with a negative input of miRNA mir-145 (see Figure 8B). In this case the classifier predicts every sample to be cancerous if mir-145 is at a low expression level. All other samples are predicted to be healthy. mir-145 is also marked as down-regulated in a study by Farazi et al. (2011). The six resulting classifiers are presented in Table 2. In the next section we discuss additional optimality criteria for choosing between several perfect classifiers.

### 3.7. Classifier evaluation

We evaluated our classifiers with the *scores.py* script and calculated the following scores: false negative rate (FN rate), false positive rate (FP rate), *S*_*AUC*_, *S*_*m*_. We run the calculations keeping the same biochemical parameter sets and binarization thresholds for each dataset as proposed by Mohammadi et al. (2017) to allow a comparison with their results. In the comparison we consider only pruned circuit designs, that is, circuits post-processed by authors to decrease the overall number of inputs. Mohammadi et al. (2017) presented in the article the following scores: *S*_*AUC*_, *S*_*m*_. Additionally, we calculated FN and FP rates for these circuits to compare the scores in the binary setting. Note that Mohammadi et al. (2017) perform the calculations in a different modeling framework and consider the discretization error as one of the optimality criteria while our classifier is only optimized on the binary dataset. Nevertheless, the central goal in both approaches is to find minimal classifiers. We present the scores in Table 2.

**Table 2:** Evaluation of breast cancer classifiers with scores: false negative rate, false positive rate, AUC, average margin and worst margin.

**Subtype**	**Classifier**	**FN rate**	**FP rate**	***S*_*AUC*_**	***S*_*m*_**
BC All	(¬ miR-378)	0.02	0.27	0.96	0.16
Triple-	(¬ miR-378) ∧ (¬ miR-144)	0.04	0.18	0.98	0.24
	(miR-24-1) ∧ (¬ miR-378)	0.04	0.18	0.99	0.25
Her2+	(miR-21) ∧ (¬ miR-451-DICER1) ∧ (¬ miR-320-RNASEN)	0.00	0.09	0.99	0.31
ER+ Her−	(miR-21)	0.00	0.27	1.00	0.50
	(miR-21) ∧ (¬ miR-320-RNASEN)	0.00	0.18	0.96	0.14
Cell Line	(¬ miR-145)	0.00	0.00	1.00	1.50
	(¬ miR-143)	0.00	0.00	1.00	1.16
	(¬ miR-199a-2-5p)	0.00	0.00	1.00	0.96
	(¬ miR-451-DICER1)	0.00	0.00	1.00	0.93
	(¬ miR-146a)	0.00	0.00	1.00	0.55
	(¬ miR-425)	0.00	0.00	1.00	0.32

For the *All* dataset Mohammadi et al. (2017) presented a classifier with scores: FN rate = 0.63, FP rate = 0.00, *S*_*AUC*_ = 1.00, *S*_*m*_ = 0.40. Our one-input classifier for this dataset is shorter (three inputs less). *S*_*m*_ shows that the margins are lower, although the FN rate is noticeably improved. The cross-validation results for the same dataset for 3-fold cross-validation presented by Mohammadi et al. are: *S*_*AUC*_ = 0.99, *S*_*m*_ = 0.31. Our results for a 3-fold cross-validation (*S*_*AUC*_ = 0.93, *S*_*m*_ = 0.24) shows that our method separated the new samples with a very similar accuracy. Note, that the samples were divided into random subsets by us and by Mohammadi et al. (2017) independently.

In case of the *Triple-* dataset we have to choose between two classifiers. Mohammadi et al. (2017) choose the best classifier by first looking at the highest *S*_*AUC*_ score and then (in case of equal values) at the highest *S*_*m*_. Based on the same strategy we chose the second classifier (*miR-21-1*) ∧ (¬ *miR-378*) as the best classifier for the *Triple-* dataset. In this case, the classification results in 5 misclassified samples in the binary setting in total. The margins for the pruned classifier for the same subset presented by Mohammadi et al. (2017) show better accuracy (FN rate = 0.10, FP rate = 0.00, *S*_*AUC*_ = 1.00, *S*_*m*_ = 0.51). However, the error rates correspond to 7 errors in total. The classifier optimized with our approach is simpler (one input less). Thus, it could be easier to assemble. Additionally, we again reduced the overall number of errors in the binary setting.

For the *Her2*+ subtype Mohammadi et al. (2017) proposed a classifier with scores: FN rate = 0.00, FP rate = 0.27, *S*_*AUC*_ = 1.00, *S*_*m*_ = 0.53. Classifier (*miR-21*) ∧ (¬ *miR-451-DICER1*) ∧ (¬ *miR-320-RNASEN*) results in only one false positive (FN rate = 0.00, FP rate = 0.09). Here, we reduced the overall number of errors from 3 to 1 in the binary setting. The lower *S*_*m*_ score is probably related to the one outlying sample captured by the FP rate. Our classifier is also shorter (two inputs less). Thus, it could be easier to assemble in the laboratory. In this case it is worth to consider which constraint is more important.

**Figure 9:**
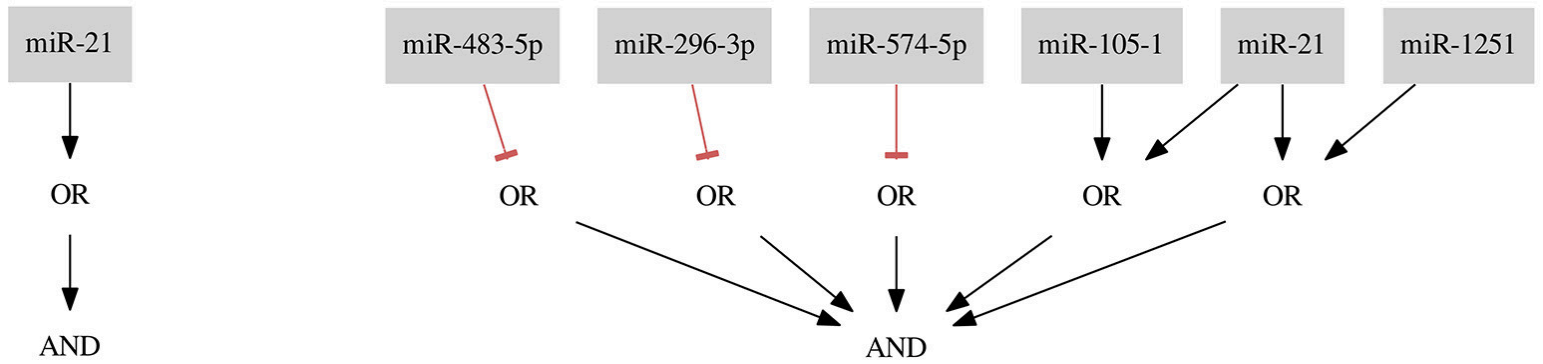
Results for the *ER*+ *Her*− data set. On the left side a classifier optimized with our approach, on the right side a classifier proposed by Mohammadi et al. (2017) The single-input classifier consists of only one miRNA which appears twice in the 7-input classifier as one of the inputs in the OR gate.

For the *ER*+ *Her*− subtype Mohammadi et al. (2017) presented a classifier with scores: FN rate = 0.00, FP rate = 0.27, *S*_*AUC*_ = 1.00, *S*_*m*_ = 0.65. Accuracy of our single-input classifier *(miR-21)* is similar and the classifier is shorter (six inputs less). Both classifiers are presented in Figure 9.

Lastly, in case of the *Cell Line* dataset all our classifiers are again shorter (two inputs less). Based on the previously described strategy we chose (¬ miR-145) as the best classifier. The scores in the continuous setting (Mohammadi et al., 2017: FN rate = 0.00, FP rate = 0.00, *S*_*AUC*_ = 1.00, *S*_*m*_ = 1.71) are comparable.

In all cases we were able to find shorter classifiers and in most cases improve the accuracy of classification in the binary setting. Otherwise, the accuracy is identical. In the continuous setting we obtained comparable results. However, interpretation of these scores linking the Boolean to the continuous classifier are difficult to assess. We address this problem in the discussion.

### 3.8. Performance analysis on simulated data

Two case studies cannot give a broad picture of the performance of an algorithm. In particular we were interested in the approximate number of samples and miRNAs at the breaking point when the ASP solver does not find a solution anymore. Clearly, the answer depends on a lot of parameters: How is the data generated? What are the constraints that specify feasible solutions? How long do we wait before a problem is deemed unsolvable?

**Figure 10:**
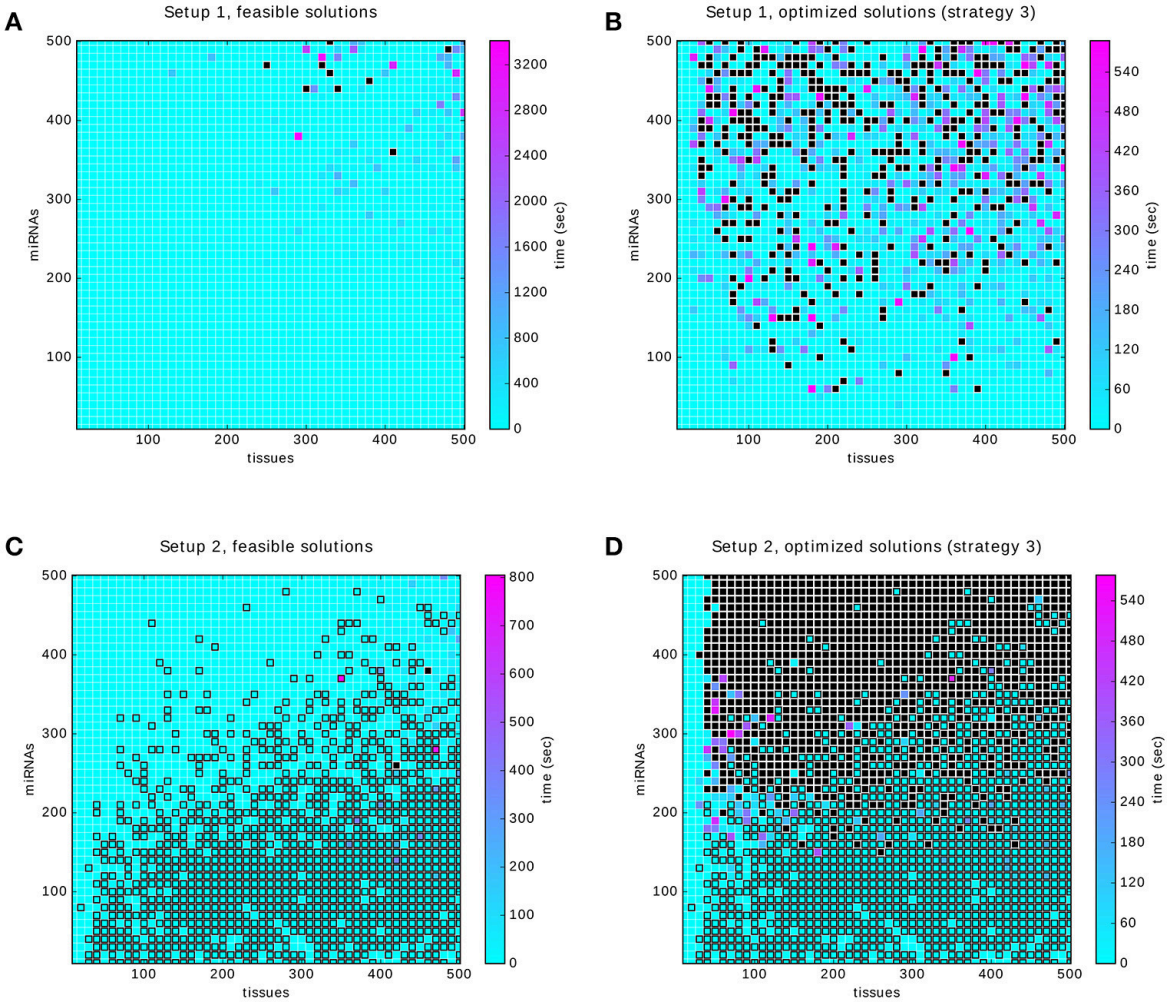
Results of the benchmarking. Black filled squares indicate that the time-out was reached. Squares outlined in black indicate that the infeasibility of the problem was proved within the time limit. **(A,B)** relate to Setup 1. They show the time to compute a feasible and an optimized solution, respectively. **(C,D)** show the times to find feasible and optimal solutions for Setup 2. In both setups the optimization strategy 3 was used (first gates, then inputs).

Each considered dataset consists of a random 0-1 matrix and an annotation that specifies which rows of the matrix correspond to positive and which to negative samples. Here, we made use of two approaches to data generation (described in detail in section 2.7): *Setup 1* which ensures that the perfect classifier exists by a controlled annotation of samples and *Setup 2* in which the annotation is generated in a less restricted manner. The crucial difference between the two setups is that Setup 1 guarantees the existence of a solution while Setup 2 does not. A solution classifier is required to satisfy the *core constraints*. We searched for feasible solutions for both setups and measured the time. The resulting four heat maps are shown in Figure 10.

To obtain the benchmark in a reasonable amount of time we used time-outs between 10 min and 1 h. Figure 10A shows that within the limits of up to 500 samples and miRNAs, a solution can be found in a reasonable amount of time. Of the 2,500 problems in Figure 10B about 16%, that is 401, were timed out. We randomly sampled 10% of these and tried solving them again with a time-out of 5 h. The result is that 27 problems could be solved, at an average of 1 h and 27 min and the remaining ones were timed-out again.

The problem of finding a feasible solution seems to increase equally with the sample and the miRNA dimension, see Figure 10A. Computing an optimal solution is, however, dominated by the number of miRNAs as can be seen in Figure 10B. We believe that this is caused by the size of the underlying search space that grows exponentially with the number of inputs, since essentially all subsets of miRNAs and possible classifiers may be optimal. Adding more samples, on the other hand, will quickly lead to redundant information since most of the input-output values of the *annotation classifier* are likely to be already covered.

In Setup 2 we marked problems that were proven to have no solution with a black outline in the heat map. Interestingly, the likelihood that a solution exists increases with the number of miRNAs, see Figures 10C,D. On the one hand, as the number of miRNAs increases, the *annotation classifier* will cover more and more gates (fixed ratio of possible gates to inputs in Setup 2) and inputs and hence it will be less likely for a classifier satisfying the *core constraints* to exist. On the other hand, each miRNA that is added beyond the ones that are actually used by the classifier has a slight chance of being exactly the input that the solution needs. Note that the expression levels of miRNAs are chosen independently and so each new input may provide a new information to the classification problem. We believe that in this setup adding miRNAs may increase the likelihood of finding a feasible solution. Here, this effect seems to be dominant, hence the infeasibility of the problem decrease while increasing the number of miRNAs. Note that this is very different from real data where miRNA expression levels can hardly be expected as independent variables (one would expect strong correlations). Figure 10D is similar to Figure 10C but the additional requirement of optimality affects the number of time-outs as the number of miRNAs is increased, see also Figure 10B.

Overall, the two plots of Setup 1 show that only a small portion of problems may not be solved, even within the time limit of up to 15 min which could be well extended in practice. The plots of Setup 2 show that the infeasibility of constraints and data can also be decided in many cases within the 15 min.

**Figure 11:**
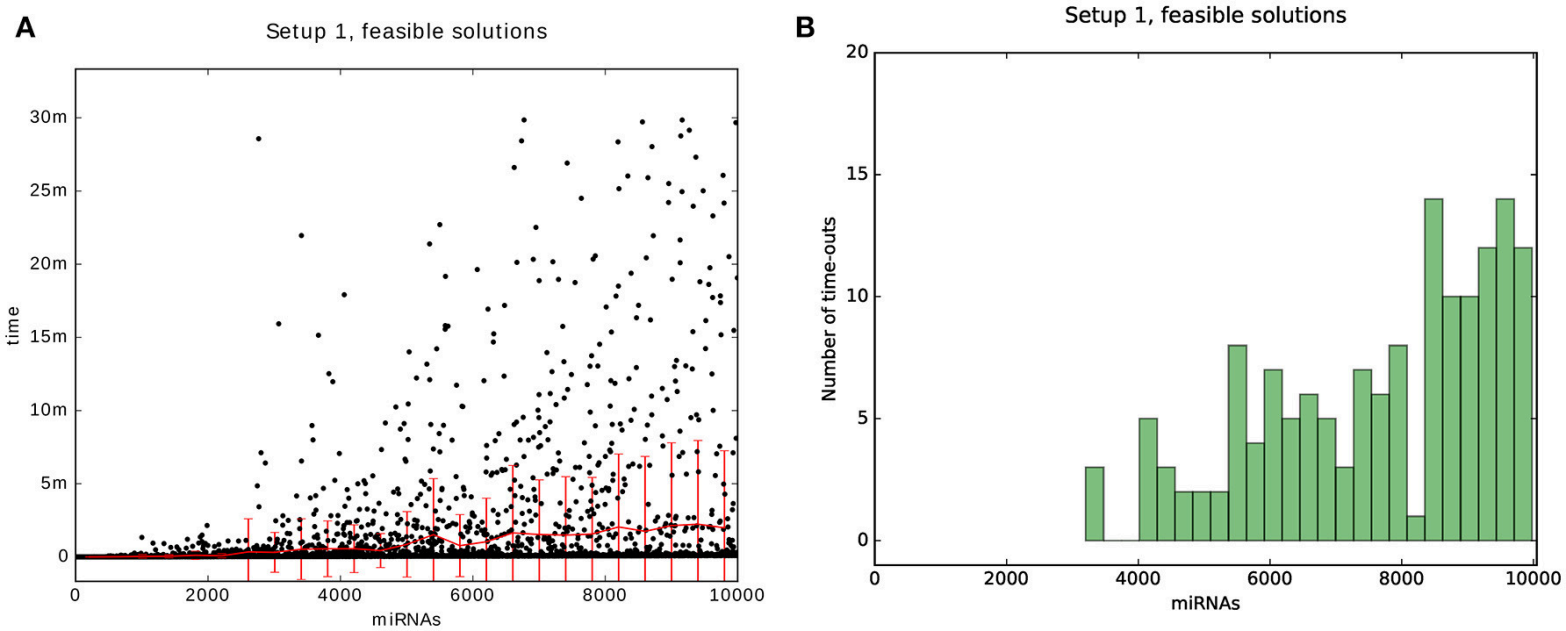
Results of the scalability test. The plot is a benchmark for 50 sampels and miRNAs that range between 50 and 10,000 with a time-out of 30 min. **(A)** is a scatter plot of the problems that could be solved within the time-limit for Setups 1. The red line and bars show the mean and standard deviation. **(B)** is a histogram of the number of problems that were timed-out.

Finally, we decided to benchmark the *scalability* of the algorithm with respect to the number of miRNAs, as we expect that in the future the data matrices will be larger in that dimension than in the sample-dimension. The benchmark can be thought of as a single column of one of the previous heat maps but with more time per problem and for up to 10,000 miRNAs. The results are shown in Figure 11. For the scalability results we sampled uniformly 4,500 data points from the 10,000 possible problems. The problems were generated with Setup 1 and with a time-out of 30 min.

We see that the mean and standard deviation are both below 10 min, even for 10,000 miRNAs. Also, the number of problems that were not solvable within the time-limit remains below 20 for a bin size of 400 problems.

Note, that for the tests we used a computing power corresponding to a power of a personal computer. We were able to find feasible solutions on a scale of minutes. When considering real-world applications one certainly may invest more time and computing power to obtain feasible, globally optimal solutions for large datasets.

### 3.9. Cross-validation

**Figure 12:**
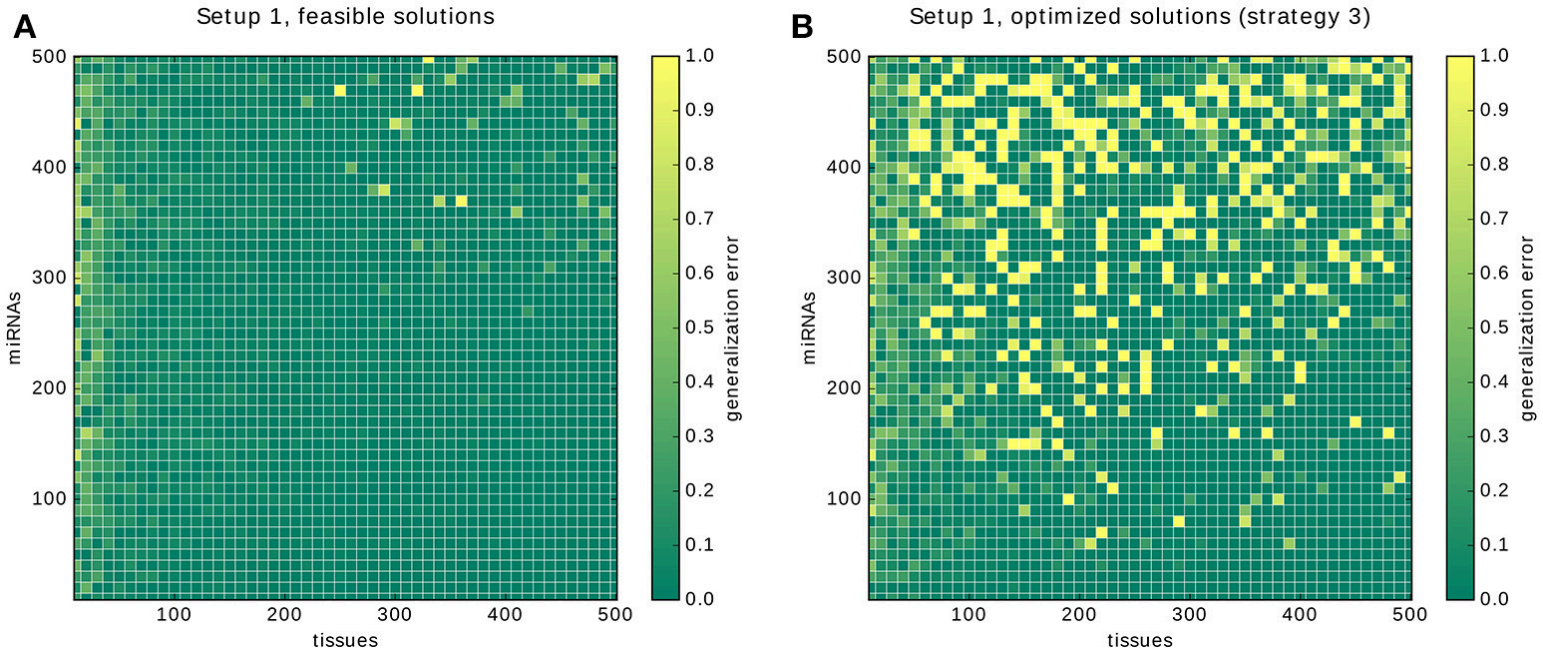
Results of the cross-validation. The results of the cross-validation for Setup 1. **(A)** shows the cross-validations for finding feasible solutions. **(B)** shows the cross-validations for finding solutions optimized with strategy 3, that is, first gates then inputs. Note that the growing error rates are due to our conservative handling of time-outs during the validation, see main text.

Here, we present the results for the 10-fold cross-validation for Setup 1 described in the previous section. The plot in Figure 12A shows two areas with high error rates: a vertical strip on the left and the circular area in the top right corner. Our interpretation for the first one is that depending on the number of gates and inputs that are used to form a classifier, a certain number of samples is required before the predictions of a solution become reliable. In the case of the *core constraints* we need around 30 samples. The likely reason is that for low sample numbers only a few input-output behaviors of the annotation are covered, leaving much room for alternative classifiers that perform poorly on the hidden data.

The circular region with an increased error in the top right corner, and in particular, the 4 data points that were assigned an error rate of 1.0, are explained by the way we deal with time-outs during the cross validation. If no feasible classifier is found during a cross-validation we count the whole tenth as false predictions. The bright yellow dots are problems in which the time-out of 10 min was insufficient for each of the 10 calculations and hence everything was counted as misclassified.

The same is happening in Figure 12B: the increased error rate along the miRNA axis reflects that the underlying problems become too hard for the time limit. Our hypothesis is that, given unlimited time and with an increasing number of samples and miRNAs, the error rates will tend toward 0. The justification is that, first, the existence of a solution is guaranteed by the setup. And second, the number of gates and inputs of the classifier is bounded by 6 and 10 respectively. Eventually, every possible input-output combination will appear in each of the 10 learning sets and the error rate will therefore tend to 0.

## 4. Discussion

The main goal of this study was to show the potential of Answer Set Programming for design problems in synthetic biology, in particular, in the context of the miRNA-based classifier design. We created a multi-step workflow for classifier optimization, which allows to obtain globally optimal perfect and imperfect (in case when a perfect classifier does not exist) classifiers in a short time using the computing power of a personal computer. The constraints we employ, that is, the gate types and bounds on inputs and occurrences reflect real life requirements for practical circuit designs (Mohammadi et al., 2017). In case when a perfect classifier does not exist, which is a common problem working with real-world data, we apply the *constraints relaxation* procedure. For the imperfect classifier we may then identify the misclassified samples, investigate the reliability of miRNA profiles for these samples and make a decision whether some of them may be neglected. On the whole, the procedure returns globally optimal solution where the main emphasis is placed on classifier accuracy followed by design simplicity in terms of number of inputs and gates. Furthermore, the ASP-based approach allows to list all optimal solutions for a given problem that the solutions can then be ranked according to lower-level criteria. Additionally, the solutions can be further analyzed to identify core features and the extent of variability.

Five real-world case studies demonstrate that the ASP-based approach allows to find shorter classifiers than heuristic methods (Mohammadi et al., 2017), when not optimizing additional cost functions. We were able to find solutions that result in lower false negative and false positive rates and decrease the overall number of errors for all presented data sets. Although we do not optimize according to the optimality criteria related to continuous cost functions as proposed by Mohammadi et al. (2017), we still achieved comparable scores in the continuous setting. These scores can also be used to further rank the classifiers in the solution set.

Unfortunately, the criteria and scores in both settings, binary and continuous, are not easily comparable and cannot be intuitively interpreted together. Future work will aim at integrating the different aspects employed in choosing the optimal classifier in the optimization criterion used for scanning the design space. Beyond the notions already explored in this paper, we plan to furthermore integrate weights representing the assessment of data quality, sensitivity to data discretization and a preference for particular circuit building blocks to foster reusability of available molecular constructs.

Although we find globally optimal feasible solutions the datasets used for the case study analysis were imbalanced. Most of them consist of several positive samples and only a few negative samples. It is worth to investigate whether the imbalanced datasets affect the results and employ additional statistical methods to decrease the possible influence in a pre-processing step.

The breast cancer datasets we considered here were pre-processed by Mohammadi et al. (2017). In the future one may extend the workflow with a data discretization pre-processing step. First of all, it may be worth to compare available methods for data discretization (Gallo et al., 2016) and employ the most suitable one for the miRNA datasets. Also, to generalize the discretization error one may consider to estimate discretization margins. The margins allow to assess the reliability of the discretization process and may help to screen out the most valuable miRNAs, that is, miRNAs of which discretized values are distant from the threshold. Discretization margins may then be used as weights for the inputs among the dataset. In other words, the weights for the particular miRNAs make them more or less desirable in the optimized classifier. Based on the weights it may be possible to incorporate an improved method of optimization based on a cost function calculated as a weighted sum for miRNA inputs used in the Boolean function. Considering the discretization error may then result in more reliable classifiers.

The benchmarks suggest that if a set of samples has a feasible solution then it can be found efficiently using ASP. That is, even for hundreds of samples and miRNAs, solutions may typically be obtained on the scale of minutes rather than hours or days using a personal computer. Thus, the benchmarks underline the feasibility of our approach for large datasets, especially in medical applications. In case of classification based on personalized miRNA profiles similar to the case studies presented in this work the ASP-based method seems to be adequate and does not require additional computational power.

We proposed a classifier design method which allows to obtain globally optimal solutions in a short time. The method is flexible in relation to the given constraints resulting from the complexity of the biological problem. We also presented several possibilities to extend the presented tool in the future. However, the task of classifier design is a complex task demanding an ongoing cooperation on both sides: experimental and computational to achieve the compromise between the biological requirements and computational possibilities.

## Data and software availability

Python scripts and pre-processed data sets used for case study analysis are available on GitHub: https://github.com/hklarner/RnaCancerClassifier.

The Potsdam Answer Set Solving Collection Potassco is available at: http://potassco.sourceforge.net/.

## Author contributions

KB, HK, and HS conceived the study. KB and HK implemented the code and prepared the first draft of the manuscript. HK designed and performed the simulated data analysis. MN extended the framework with the evaluation procedure, performed the case studies and wrote the final version of the manuscript in consultation with KB, HK, and HS. HS supervised the project.

### Conflict of interest statement

The authors declare that the research was conducted in the absence of any commercial or financial relationships that could be construed as a potential conflict of interest.
