# Supplementary material for: Designing miRNA-Based Synthetic Cell Classifier Circuits Using Answer Set Programming
Source: Front Bioeng Biotechnol. 2018 Jun 22;6:70. doi: 10.3389/fbioe.2018.00070 (PMC6023966; doi:10.3389/fbioe.2018.00070)
Supplement: Supplementary file 1 [file Presentation_1.PDF]

Supplementary Material: Designing miRNA-based Cell Classifiers Using Answer Set Programming

1 SUPPLEMENTARY DATA

1.1 Introduction to Answer Set Programming (ASP)

Answer Set Programming (ASP) is a form of declarative programming and based on the stable model semantics of logic programming.

DEFINITION 1 (Reduct). *The* reduct P^X *of a program* P *relative to a set* X *of atoms is defined by*

$$P^X = \{head(r) \leftarrow body^+(r) \mid r \in P \text{ and } body^-(r) \cap X = \emptyset\},$$

where $body^+(r)$ *is the set of all positive atoms of the body and* $body^-(r)$ *is the set of all negative atoms of the body.*

DEFINITION 2 (Stable Model). *A set* X *of atoms is a* stable model *of a program* P *if the inclusion-wise minimal model of the reduct* P^X *of* P *relative to* X *is equal to* X.

Problems are formulated as logic programs which are finite sets of rules. A *rule* r is of the form

$$A_0 \text{ :- } A_1, \ldots, A_m, \text{not } A_{m+1}, \ldots, \text{not } A_n, \tag{S1}$$

where $n \geq m \geq 0$, each A_i, $0 \leq i \leq n$, is an *atom* and 'not' stands for negation by default.

To find a solution the problem representation is first passed to an ASP grounder which replaces the variables in rules by constants in ground instances. The grounded program is then forwarded to an ASP solver which computes the stable models of the program.

In the following, we explain the input language used by Potsdam Answer Set Solving Collection *Potassco*. A rule is a conditional constraint, which means that the head, the left hand side of the rule, must be true if the body, the right hand side of the rule, is true. If $n = 0$, rule (S1) is called a *fact*, meaning that the head of the rule is always true. If a rule has an empty head, it represents an *integrity constraint*. Such a rule expresses that a stable model must not satisfy the body. Therefore integrity constraints are often used to eliminate model candidates of a program. Rules can also include *conditinal literals* which are of the form $A : B_1, \ldots, B_m$ where A and B_i are possibly default negated literals for $0 \leq i \leq m$. Such conditional literals can then be used to construct *cardinality constraints* which can be written as $s\ \{C_1; \ldots; C_n\}\ t$ where each C_j is a conditional literal for $1 \leq i \leq n$ and $s, t \in \mathbb{N}$ provide lower and upper bounds on the number of satisfied literals.

The input language of ASP allows to do optimization. To minimize the sum of weights w_i of literals B_i the user writes `#minimize` $\{w_1 : B_1; \ldots; w_n : B_n\}$. For a more detailed description of the input language of the Potassco ASP read the user's guide (See section Data and Software Availability).

1.2 The ASP encoding

1.2.1 User input

1.2.1.1 Sample data

In a first step the input data is converted into so-called facts, predicates that are specified to be true and which the solver uses to deduce the values of other predicates. The predicate `sample` specifies whether a sample of a given index is `healthy` or `cancer`ous. Note that lower-case words are constants in the Potassco ASP input language. The predicate `data` contains information about the miRNAs expression level in each sample. The first variable is the sample index, the second one is the name of the miRNA and the third one is either `high` or `low` depending on whether the miRNA expression was binarized to 1 or 0. We believe that the use of character constants as arguments to predicates, instead of only numbers, increases the overall readability of the program.

```
sample(1,healthy). sample(2,healthy). sample(3,cancer).

data(1,g1,high). data(1,g2,high). data(1,g3,low).
data(2,g1,low). data(2,g2,low). data(2,g3,high).
data(3,g1,low). data(3,g2,high). data(3,g3,low).
```

The constraints in the sections below frequently need to iterate over all samples, or over all miRNA. To be able to reference all existing sample indices, or all existing miRNA indices we must use so-called variable binding via two additional predicates `is_sample` and `is_mirna`.

```
is_sample_id(X) :- sample(X,Y).
is_mirna(Y) :- data(X,Y,Z).
```

1.2.1.2 Gate types and inputs

Like the sample data, the input constraints are defined in terms of facts. The first two lines specify the bounds on the total number of miRNA inputs and the number of gates used in the classifier.

```
lower_bound_inputs(1). upper_bound_inputs(10).
lower_bound_gates(1). upper_bound_gates(2).
```

Next the *gate types* that can be used in the classifier are defined. The predicate `is_gate_type` assigns an index to a gate type and is also used for variable binding. Then the bounds on positive and negative input miRNAs to the gate type are given, each bound to the gate type via the corresponding index. The last fact concerning the gate type is the upper bound on its occurrence.

```
is_gate_type(1).
lower_bound_pos_inputs(1, 0). upper_bound_pos_inputs(1, 2).
lower_bound_neg_inputs(1, 0). upper_bound_neg_inputs(1, 0).
upper_bound_gate_occurence(1, 1).
```

Encoding of feasible classifiers

1.2.1.3 Gates

Our approach to encoding feasible classifiers follows the *generate-and-test* principle of Potassco ASP. We create the predicate `number_of_gates` with an integer argument that specifies how many gates exist in the current solution. The 1 before and after the curly brackets enforce that `number_of_gates` is true for exactly one input between the lower and upper bounds specified by the user. As before, we create a predicate `is_gate_id` for referencing all indices of existing gates, i.e., between 1 and the number of gates.

```
1 {number_of_gates(X..Y)} 1 :- lower_bound_gates(X), upper_bound_gates(Y).
is_gate_id(1..X) :- number_of_gates(X).
```

Now we assign a gate type to each existing gate via the predicate `gate_type` which takes to arguemnts, a gate index and a gate type index. Note that here is the first instance of iterating over existing values via some of the variables bindings from above. We require that each gate index is assigned to exactly one gate type index.

```
1 {gate_type(GateID, X): is_gate_type(X)} 1 :- is_gate_id(GateID).
```

The next step is to ensure that the number of positive and negative inputs to each gate is consistent with the bounds specified by the user for the respective gate type. Hence, we create a predicate `gate_input` that assigns a miRNA of a certain sign to a gate. We use `positive` and `negative` to represent non-negated and negated inputs to gates. Both are accessible via the predicate `is_sign`.

```
X {gate_input(GateID, positive, MiRNA): is_mirna(MiRNA)} Y
   :- gate_type(GateID, GateType),
   lower_bound_pos_inputs(GateType, X), upper_bound_pos_inputs(GateType, Y).
   is_sign(positive). is_sign(negative).
```

Note that this constraint allows that an input appears negated and non-negated in the same gate, resulting in a gate that fires (see below) whether the input is present or not, i.e., a gate that is always active. Mathematically it is not useful to add a constant-1 clause to a CNF. To avoid such gates we could introduce the following additional count constraint:

```
{gate_input(GateID, Sign, MiRNA): is_sign(Sign)} 1
    :- is_mirna(MiRNA), is_gate_id(GateID).
```

But, according to Mohammadi et al. (2017), we are frequently interested in classifiers whose inputs are unique across all gates. In such cases we may drop this constraint and use the unique-inputs-constraint (see below).

The number of times a gate of a certain type appears in a solution is constrained by the upper bound on the gate occurrences:

```
{gate_type(GateID,GateType): is_gate_id(GateID)} X
   :- upper_bound_gate_occurence(GateType,X).
```

1.2.1.4 Inputs

It may be that a gate type is defined with both lower bounds of positive and negative inputs set to 0. In that case the solver may choose to create a gate with no inputs at all. Since solutions that contain gates with no inputs are mathematically and biologically meaningless we add a constraint that says that the overall count of inputs to a gate must be greater than 1:

```
1 {gate_input(GateID,Sign,MiRNA): is_sign(Sign), is_mirna(MiRNA)}
   :- is_gate_id(GateID).
```

It seems that unique inputs are frequently desired (across the whole classifier), see Mohammadi et al. (2017). Hence we added a unique-inputs-constraint that requires that each miRNA (negated or not) appears in at most one gate.

```
{gate_input(GateID,Sign,MiRNA): is_sign(Sign), is_gate_id(GateID)} 1
   :- is_mirna(MiRNA).
```

If this constraint is dropped then it should still be useful to require that an input does not appear in its negated and non-negated form in the same gate (see above). To constrain the overall number of inputs that appear in the classifier we need an additional cardinality constraint.

```
X {gate_input(GateID,Sign,MiRNA):
   is_gate_id(GateID), is_sign(Sign), is_mirna(MiRNA)} Y
   :- lower_bound_inputs(X), upper_bound_inputs(Y).
```

1.2.1.5 Firing of gates

To evaluate how the classifier responds to a sample, the reaction of the individual gates is examined first. A gate fires for a given sample, if the expression of a miRNA (`high` or `low`), which is used as an input for the gate, is consistent with the sign of the input (`positive` or `negative`).

```
gate_fires(GateID,SampleID)
   :- gate_input(GateID,positive,MiRNA), data(SampleID,MiRNA,high).

gate_fires(GateID,SampleID)
   :- gate_input(GateID,negative,MiRNA), data(SampleID,MiRNA,low).
```

1.2.1.6 Predicition of the classifier

The prediction of the classifier is then generated using the facts `gate_fires`. As the classifiers we are looking at are in *conjunctive normal form* (CNF), the classifier only evaluates to 1, i.e., *predicts cancer* iff all gates fire. This means, that if any of the gates does not fire, the classifier will predict the issue to be `healthy`. In all other cases it will predict `cancer`.

```
classifier(SampleID,healthy)
   :- not gate_fires(GateID, SampleID), is_gate_id(GateID),
   is_sample_id(SampleID).
```

```
classifier(SampleID,cancer)
   :- not classifier(SampleID, healthy), is_sample_id(SampleID).
```

1.2.1.7 Consistency of classifier and data

We use two integrity constraints to guarantee, that the classifier is agreeing with the data, meaning that it is *not* a valid answer set, if the sample is assigned to be `healthy` and the classifier predicts `cancer` or the other way around.

```
:- sample(SampleID,healthy), classifier(SampleID,cancer).
:- sample(SampleID,cancer),  classifier(SampleID,healthy).
```

DATA AND SOFTWARE AVAILABILITY

Python scripts and pre-processed data sets used for case study analysis are available on GitHub: `http://github.com/hklarner/RnaCancerClassifier/blob/master/classifier.py`.

The Potsdam Answer Set Solving Collection *Potassco* is available at: `http://http://potassco.sourceforge.net/`.

REFERENCES

Mohammadi, P., Beerenwinkel, N., and Benenson, Y. (2017). Automated design of synthetic cell classifier circuits using a two-step optimization strategy. *Cell Systems* 4, 207–218. doi: 110.1016/j.cels.2017.01.003
